# Adulthood Psychosocial Disadvantages and Risk of Hypertension in U.S. Workers: Effect Modification by Adverse Childhood Experiences

**DOI:** 10.3390/life12101507

**Published:** 2022-09-27

**Authors:** Timothy A. Matthews, Yifang Zhu, Wendie Robbins, Mary Rezk-Hanna, Paul M. Macey, Yeonsu Song, Jian Li

**Affiliations:** 1 Department of Environmental Health Sciences, Fielding School of Public Health, University of California Los Angeles, Los Angeles, CA 90095, USA; 2 School of Nursing, University of California Los Angeles, Los Angeles, CA 90095, USA; 3 David Geffen School of Medicine, University of California Los Angeles, Los Angeles, CA 90095, USA; 4 Geriatric Research Education and Clinical Center, Veterans Affairs Greater Los Angeles Healthcare System, Los Angeles, CA 90095, USA

**Keywords:** psychosocial stress, childhood adversities, job strain, social isolation, hypertension

## Abstract

Hypertension is a key driver of cardiovascular diseases. However, how stressors contribute to the development of hypertension remains unclear. The objective of this study was to examine prospective associations of adverse childhood experiences (ACEs) and adulthood psychosocial disadvantages (APDs) with incident hypertension. Data were from the Mid-life in the United States (MIDUS) study, a national, population-based, prospective cohort study. ACEs were examined via retrospective reports, and APDs including work stress and social isolation were assessed using survey measures. Incident hypertension was defined based on self-reported physician diagnosis. Baseline data were collected in 1995, with follow-up in 2004–2006 and 2013–2014. Cox proportional hazards regression was applied to assess prospective associations of ACEs and APDs with incident hypertension in 2568 workers free from hypertension at baseline. After adjustment for covariates, baseline APDs were associated with increased incident hypertension (aHR and 95% CI = 1.48 [1.09, 2.01]) during a 20-year follow-up, whereas ACEs showed null associations. Moreover, a moderating effect by ACEs was observed—the effect of APDs on risk of hypertension was stronger when ACEs were present (aHR and 95% CI = 1.83 [1.17, 2.86]). These findings underscore the importance of psychosocial stressors as nontraditional risk factors of cardiometabolic disorders.

## 1. Introduction

Hypertension is a ubiquitous and pressing issue of major public health significance due to its role as a major driver of cardiovascular diseases (CVD, including coronary heart disease and stroke), which are the leading causes of death and disability in the United States (U.S.) and globally [1]. While cardiovascular disease incidence has decreased among older adults in the past few decades, current evidence has underscored a contrasting trend of increased cardiovascular disease burden in working populations, especially among younger individuals [2]. Furthermore, recent data indicate an overall decline in working-age mortality rates among most economically developed nations since 2010, whereas in the U.S., mortality rates for working populations have shown the opposite pattern, worsening over time due especially to hypertensive heart disease [3].

Past attempts to ameliorate the epidemic of hypertension have predominantly emphasized traditional risk factors such as smoking, diet, and exercise [4]. More recent evidence has identified a key role of non-traditional risk factors such as psychosocial stressors as critical elements of hypertension etiology [5]. Among these psychosocial factors, work stress and social isolation have received special attention [6,7,8,9,10,11]. Job strain, a well-established operationalization of work stress, has reliably been associated with hypertension in systematic reviews and meta-analyses [6,7]. Social isolation, defined as a lack of social contacts and shortage of social relationships, has been evidenced as a severe psychosocial stressor in adulthood that demonstrates robust associations with CVD and consistently predicts increased hypertension risk [8,9,10,11].

In line with previous attempts to assess the interplay of psychosocial stressors across childhood and adulthood [12,13,14], we include in our exposure models adverse childhood experiences (ACEs, e.g., parental abuse/neglect) as indicators of early life stress, and both job strain and social isolation as metrics of work and nonwork related stress in mid-life. To our knowledge, only few studies based solely on European cohorts have attempted to investigate potential interactions between ACEs, work-related stress, and adulthood health, reporting mixed and inconsistent findings, which, along with no data from the, U.S. present a gap in knowledge [15,16,17,18,19,20]. Importantly, the majority of previous studies assessing psychosocial stressors separated work and non-work-related factors when investigating associations with hypertension; notably, one Canadian study found that the combination of work stress and social stress improved risk estimates for high blood pressure over a 5-year follow-up period [21]. Therefore, cumulative adulthood stress deserves further exploration, and hence, we combine job strain and social isolation to form the construct of adulthood psychosocial disadvantages (APDs) as an index of cumulative adulthood stress. Our analytic approach was designed to elicit the relative contributions of psychosocial exposures to cardiometabolic disease burden across the life course, examining ACEs in childhood and positing the construct of APDs in mid-life adulthood.

The overall objective of this study was to study associations of work and non-work-related psychosocial stressors with incident hypertension, using data from the national, population-based Mid-life in the United States (MIDUS) [22,23] study with prospective cohort design. Furthermore, while prior research has suggested that APDs act as mediators between associations of ACEs and adulthood health outcomes [14], another line of evidence has argued that ACEs in fact act as effect modifiers, moderating associations of adulthood stress with disease [15,16]. Therefore, our aims were two-fold: first, to assess prospective associations of ACEs and APDs with incident hypertension; and second, to examine effect modification of associations between APDs and hypertension by ACEs exposure. We hypothesize that higher ACEs and APDs at baseline exposures will be associated with higher risk of incident hypertension, compared to lower exposure levels, and that incident hypertension risk will be higher when both ACEs and APDs are present.

## 2. Materials and Methods

### 2.1. Sample Population

**Figure 1:**
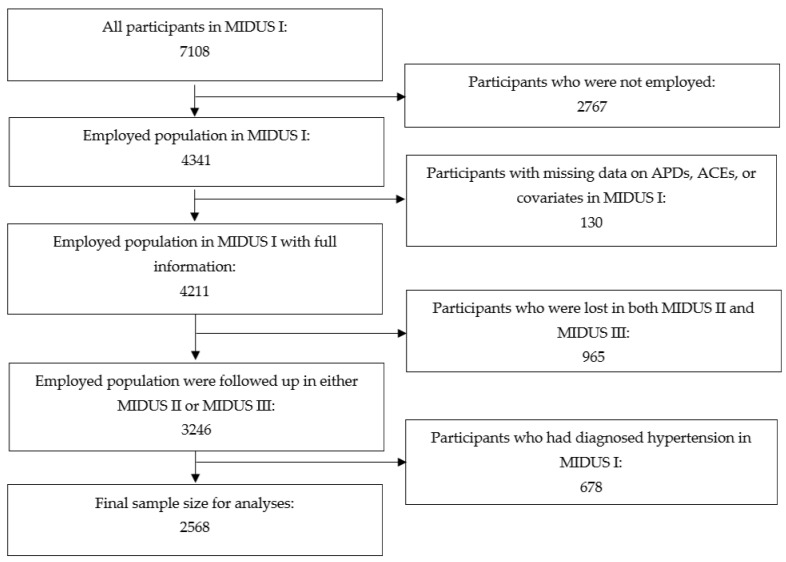
Sample Size Selection Flowchart.

Data for this study were drawn from the MIDUS I, II, and III surveys. The MIDUS surveys were national, population-based cohort studies of psychosocial, behavioral, and health factors in U.S. adults. The MIDUS surveys were conducted via random digit dial (RDD) interviews and a self-administered questionnaire (SAQ). The MIDUS study began in 1995 [22], with follow-up in MIDUS II from 2004 to 2006 [24] and in MIDUS III from 2013 to 2014 [23], culminating in maximally 20 years of follow-up time. At MIDUS I, which was the baseline time-point for the present study, there were 7108 participants, of which 4341 were employed. Among employed participants, 4211 (97%) had full data for variables included in the analyses of the current study. After the two subsequent surveys of MIDUS II and III, there were 3246 participants who were followed up at least once, representing a follow-up rate of 77%. We excluded participants with self-reported physician-diagnosed hypertension at baseline to produce accurate estimates of incident hypertension during follow-up and minimize reverse causation. The process of sample selection yielded a final sample size of 2568 participants (see Figure 1). Follow-up time began upon enrollment in the MIDUS I survey, and censoring based on hypertension incidence occurred between MIDUS I and MIDUS III. We adhered to the Strengthening the Reporting of Observational Studies in Epidemiology (STROBE) guidelines. All participants provided written informed consent. This study was reviewed and approved for exemption by the University of California, Los Angeles Institutional Review Board (IRB#22-000604).

### 2.2. Materials and Measures

Exposure measures for ACEs and APDs were derived from SAQ responses. ACEs were assessed retrospectively at baseline in MIDUS I via a series of detailed questions about the participant’s childhood, relationships with parents, and socioeconomic status in early life. These items have been used to assess associations of ACEs with adulthood disease in prior analyses of the MIDUS dataset [25]. Previous evidence examining the health impacts of ACEs have identified a factorial structure with three key subdomains of (i) parental abuse or neglect, (ii) financial stress, and (iii) household dysfunction; this compartmentalization outlines the fundamental aspects of ACEs and offers a practical methodological framework for their analysis [26,27]. The questionnaire items assessing ACEs in the MIDUS study cover the three factors, with eight items assessing parental abuse (example item: “During your childhood, how often did your mother, or the woman who raised you, hit you?”), four items measuring financial stress (example item: “During your childhood and adolescence, was there ever a period of six months or more when your family was on welfare?”), and 12 items examining household dysfunction (example item: “Did your biological mother or father die?”). A sum score for the three factor ACEs structure was calculated, and a binary ACEs variable was created by dichotomizing at the upper tertile of the sum score. Participants who experienced two or more components of the three-factor ACEs structure were classified as having high ACEs exposure, creating a dichotomous ACEs variable with categories for high and low.

APDs include job strain and social isolation. Job strain was defined according to Karasek’s demand–control model, which posits job strain as the combination of high job demands with low job control [28]. In MIDUS I (baseline), job demands were examined using five items (example item: “How often do you have to work intensively?”). Job control was assessed with nine items (example items: “how often do you learn new things at work?” “How often do you have a choice in deciding how you do your tasks at work?”). Responses for job demands and job control were recorded according to a 5-point Likert scale (1 = never, 5 = all of the time). The questionnaire items for job demands and job control in the MIDUS study are closely similar to those seen in the standard Job Content Questionnaire developed by Karasek [29] and have been used in previous publications using the MIDUS study data [30,31]. Job demands and control were dichotomized into high and low groups by their median scores (16 and 34, respectively), and therefore binary job strain was defined as combined high job demands and low job control [29]. Social isolation at baseline in MIDUS I was operationalized as per the Berkman–Syme Social Network index [32], which assesses an individuals’ degree of social connectedness via the objective indicators of frequency of social contacts and living status. The Berkman–Syme Social Network index is a well-validated and widely used measure that has been successfully applied in prior analyses of the MIDUS data [14]. The questions identify whether participants have regular contact with family members, friends (in person, on the phone, or in writing/email), a social organization, club, or group, and if they live alone, yielding a sum score for social connectedness ranging from zero to four. Participants whose sum score was less than the upper tertile (i.e., three) were categorized as socially isolated, generating a dichotomous variable with groups for high and low social isolation. APDs were defined as the combination of high job strain and/or high social isolation, resulting in a categorical variable with three levels—low (no disadvantages), moderate (one disadvantage, either high job strain or high social isolation), and high (two disadvantages, both high job strain and high social isolation).

### 2.3. Outcome

Incident hypertension (yes or no) during follow-up across the MIDUS II and III surveys was defined based on self-reported physician-diagnosed hypertension. Affirmative responses to the question, “Has a doctor ever told you that you have or had high blood pressure?”, were counted as instances of hypertension onset. The timing of hypertension onset in years since the baseline survey was self-reported via the question: “How many years ago were you told you have or had high blood pressure?” at follow-up surveys [33]. This methodological approach towards the identification of incident hypertension is in line with that of other national studies in the U.S., such as the Health and Retirement Study [34].

### 2.4. Covariates

Information on sociodemographic factors and health-related behaviors was assessed at baseline, including age (continuous), sex, race (White; Black; and Other), educational attainment (high school or less; some college; university degree or more), annual household income (<USD 45,000; USD 45,000–89,999; USD 90,000+), current cigarette smoking (yes; and no), alcohol consumption (low or moderate drinking—up to two drinks per day for men and one drink per day for women; and heavy drinking—more than moderate drinking [35,36]), and frequency of physical exercise (low—never; moderate—once a week to once a month; high—several times a week) [37]. Major depressive episode (MDE) in the past year was additionally included due to its role as a potential risk factor for hypertension [30,38].

### 2.5. Statistical Analysis

**Figure A1:**
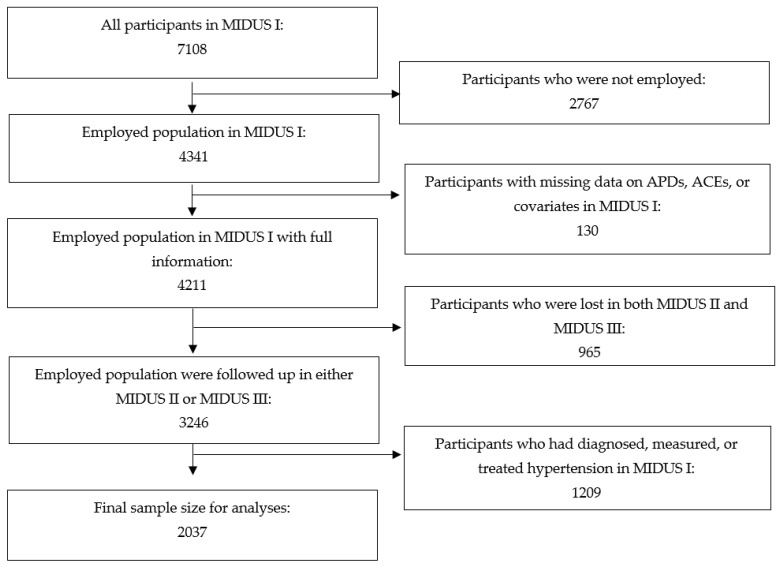
Sample Size Selection.

Data used in the analyses were collected from 1995 to 2014. First, descriptive statistics were generated, and relative frequencies were examined for characteristics of the study sample at baseline. Second, the prospective associations of ACEs, APDs at baseline were assessed separately with incident hypertension during follow-up using Cox proportional hazards regression, and the results were expressed as hazard ratios (HRs) with 95% confidence intervals (CIs). Multivariable models were calculated in four steps after unadjusted Model 0: Model I adjusted for age and sex; Model II included further adjustment for race, educational attainment, and annual household income; Model III additionally adjusted for current smoking, alcohol consumption, and physical exercise; and Model IV added adjustment for MDE. Hypothesis tests were two-sided at the 5% α level. We tested for interaction between ACEs and APDs with hypertension as the outcome, and further stratified analyses were conducted to assess effect modification by ACEs on associations between APDs and incident hypertension. Finally, we also implemented sensitivity analyses using a stricter and more inclusive definition of baseline hypertension, where prevalent hypertension was defined as either self-reported physician-diagnosed hypertension, measured hypertension (as defined by the ACC/AHA 2017 guideline, namely systolic blood pressure at least 130 mm Hg or diastolic blood pressure at least 80 mm Hg) [39], or medication-treated hypertension in MIDUS I. This approach excluded an additional 531 participants, yielding an analytic sample of 2037 (Figure A1 in the Appendix A). The SAS PHREG procedure and ASSESS function with the PH option (the supremum test) were used to verify the proportional hazards assumptions of the Cox models (*p* > 0.20). All statistical analyses were conducted using the program SAS 9.4 (SAS Institute, Cary, NC, USA).

## 3. Results

### 3.1. Participant Characteristics

The characteristics of the sample population at MIDUS I, baseline, are shown in Table 1. The sample of 2568 participants was made up of middle-aged adults with a mean age of 43 who were mostly White and had at least some college education, with approximately equal numbers of men and women. The majority of participants had an annual household income above USD 45,000 and were non-smokers, who reported low to moderate drinking, moderate to high physical activity, and were free from MDE. The prevalence of moderate to high APDs was approximately 33%, while 39% of participants reported a high level of ACEs.

### 3.2. Associations of Adverse Childhood Experiences and Adulthood Psychosocial Disadvantages at Baseline with Risk of Hypertension

During 34,993 person years of follow-up time across a 20-year follow-up period, 934 cases of incident hypertension were reported, representing an overall hypertension incidence rate of 26.69 per 1000 person years. Incidence rates for hypertension were 25.53, 28.14, and 37.40 per 1000 persons among participants with low, moderate, and high levels of baseline APDs, respectively. The results of the Cox proportional hazards regression analyses for the entire sample are presented in Table 2. While no significant associations of incident hypertension with ACEs reported at baseline were observed, a high level of APDs was significantly associated with incident hypertension (fully adjusted HR and 95% CI = 1.48 [1.09, 2.01]), compared to low levels of APDs.

### 3.3. Effect Modification of Adverse Childhood Experiences

We observed a significant interaction term between ACEs and APDs with hypertension incidence as the outcome (*p* < 0.05). Table 3 displays the results of the stratified analyses investigating effect modification of ACEs on associations between APDs and incident hypertension. The analyses indicate that for participants with low levels of ACEs, exposure to APDs was not a significant predictor of incident hypertension, while among those with high ACEs, both moderate and high levels of APDs were significantly associated with incident hypertension (fully adjusted HR and 95% CI = 1.27 [1.01, 1.60] and 1.83 [1.17, 2.86], respectively), compared to low levels of APDs.

### 3.4. Sensitivity Analyses

The results of the sensitivity analyses are exhibited in Table A1, Table A2 and Table A3 in the Appendix A. When expanding the definition of prevalent hypertension at baseline to include participants with measured high blood pressure or antihypertensive medication use, the pattern of associations remained the same, with a slight increase in effect size. Compared to those with low APDs, the risk of incident hypertension was significantly elevated among participants with high APDs (fully adjusted HR and 95% CI = 1.61 [1.15, 2.26]). Similarly, ACEs were not associated with risk of hypertension, but obvious effect modification by ACEs was observed.

## 4. Discussion

This was the first study to assess prospective associations of ACEs and APDs at baseline with incident hypertension in a national, population-based cohort of U.S. workers. Detailed information about the participants’ early life experiences was used to measure ACEs, and a measure of APDs was constructed by combining a classic measure of job strain based on Karasek’s demand–control model [29] with a well-validated measure of social isolation known as the Berkman–Syme Social Network Index [32]. While early life exposure to ACEs was not associated with incident hypertension, we found that exposure to APDs at baseline was associated with a significantly elevated risk of incident hypertension within 20 years of follow-up. These results suggest a pathological influence of psychosocial stressors in the etiology of hypertension. Our hypotheses were therefore partially supported by the findings.

These results are consistent with the literature on job strain and social isolation—the individual constituents of exposure to APDs—which has demonstrated robust and stable associations with cardiovascular diseases and hypertension [5,6,7,8,9,10,11,40]. Indeed, social isolation in adulthood has been linked to drastically increased CVD mortality risk, as well as hypertension [8,9,11]. Similarly, job strain is well-established as a major contributor to hypertension, with an extensive body of evidence demonstrating consistent and robust associations [6,7].

In addition to adulthood stressors, emerging evidence in recent years has revealed that exposure to early life adversity, or ACEs, can have pronounced deleterious impacts on multiple adult cardiometabolic health outcomes, including hypertension [12,13]. Life-course exposure models have indicated that early childhood is a critical period that greatly influences responses to environmental stressors later in life [41,42]. The present findings regarding ACEs contrast with the prevailing literature documenting the adverse impacts of ACEs on a variety of health conditions. These inconsistencies may be in part explained by the restriction of our sample to the working population, given that most studies on ACEs and adulthood cardiometabolic diseases are in the general population, especially ageing people [12,13]. In addition, due to the lack of direct associations of ACEs and risk of hypertension in our study, according to the traditional assumptions of mediation analysis, it is unlikely that a mediating effect by other variables (such as APDs) was present [43]. Nevertheless, the stratified analyses demonstrate effect modification of ACEs on associations of APDs with incident hypertension. In other words, the hypertension risk associated with APDs exposure was significantly higher among participants with higher exposure to ACEs, compared to those with lower exposure to ACEs. Such examples of moderating effects of ACEs have been previously substantiated [15,16]. For instance, a prospective cohort study of Finnish employees followed from childhood to adulthood reported that while job demands—a fundamental component of Karasek’s job strain model—predicted depressive symptoms across 6 years, this association was moderated by ACEs [15]. The study found that participants with three or more ACEs were more susceptible to developing depressive symptoms elicited by high job demands, compared to those with fewer ACEs. Similarly, a Swedish cohort study showed effect modification by early life adversity, wherein associations of job strain with increased allostatic load were detected only among participants who had experienced adversity in adolescence [16]. However, another Finnish study and an analysis based on the Whitehall II study found that pre-employment factors such as early life adversity did not moderate associations of job strain with CVD [19,20].

The findings of the stratified analyses are consistent with the vulnerability hypothesis, which emphasizes differential susceptibility to adversity between individuals based on genetic factors such as vulnerability and risk alleles, and environmental influences [44]. Potential explanations underlying observed increases in stress vulnerability with ACEs exposure include heightened systemic responses to stressors [45] and the increased appraisal of hostile intent in social interactions with others, which is likely to contribute to social isolation [46]. Evidence also indicates that ACEs may lead to more adverse working conditions and augment perceptions of stressful work environments in adulthood [47]. Furthermore, childhood adversity has been explicitly and mechanistically linked to adulthood hypertension risk, with proinflammatory mediators and vasoactive factors identified as principal biological drivers of hypertensive pathogenesis [13]. The preponderance of evidence indicates that early life adversity shapes and characterizes both autonomic physiological and psychological stress responses, exacerbating the impacts of adulthood stressors [48].

The overall findings regarding exposure to ACEs, APDs, and increased hypertension risk are biologically plausible and mechanistically sound, as the pathways underlying associations of psychosocial stressors with cardiometabolic health conditions have been clearly delineated. Encompassing the entirety of the autonomic nervous system, mechanisms involved in chronic stress response include heightened sympathetic arousal such as increased heart rate and blood pressure, neuroendocrine changes such as increased secretion of cortisol and noradrenaline, and the diffuse perturbation of the hypothalamic-pituitary-adrenal axis [49,50]. ACEs specifically have also been found to impair stress reactivity and regulation, with one major consequence being dampened cardiometabolic responses to stress, ultimately leading to increased chronic disease susceptibility [48]. Cumulatively, these pathways constitute allostatic load, a measure of wear and tear of the body due to stress demands [51]. With chronic exposures over many years, high levels of psychosocial stress from combined ACEs and APDs are likely to increase allostatic load and hence result in persistent cardiovascular burden.

### 4.1. Strengths

The major strengths of this study come from the sample population and well-validated measures used. The MIDUS study sample is large and highly diverse, including participants across a range of demographics and occupations, and has a long follow-up length of 20 years. The exposure measure of job strain was based on the well-established Karasek’s demand–control model [28], and the similarly well-validated Berkman–Syme Social Network Index was used to assess social isolation [32]. We also accounted for several important confounders and risk factors for hypertension in our multivariable analyses, including smoking, alcohol consumption, physical activity, and MDE [4,37]. Furthermore, the sensitivity analyses using an expanded definition of baseline hypertension demonstrated not only the same pattern of associations but an increase in effect size, increasing confidence in the robustness and stability of the results.

### 4.2. Limitations

There are several limitations in this study. While the reliability of adult retrospective reports of ACEs has been questioned in the past due to potential recall bias, evidence suggests that retrospective reports are generally valid, with robust test–retest reliability ranging from 0.45 to 0.90, and adequate kappa coefficients ranging from 0.52 to 0.72 [52,53]. In a similar vein, all exposure information was collected at baseline, and hence our results may be impacted by exposure misclassification bias due to potential changes in APDs during follow-up. Another limitation is the use of self-reported hypertension as the outcome, as opposed to clinically observed hypertension; however, self-reported hypertension has been shown to have good validity, particularly in large-scale epidemiological studies [54]. Additionally, our study participants were middle aged, predominantly Whites, and had higher levels of education than average levels in the U.S. Therefore, our findings cannot be generalized to those who are non-Whites, younger or older adults, and have lower levels of education. We also did not include other factors which may impact adulthood psychosocial stress furthermore risk of hypertension. For example, female workers with family responsibilities may be more vulnerable to psychosocial stress and negative physical health outcomes than counterparts without such responsibilities. Furthermore, our results may be affected by selection bias, as a substantial number of participants were lost to follow-up from MIDUS I to MIDUS III, and those impacted by attrition may have been systematically different from those who were followed up. The 965 participants lost to follow-up were more likely to be socially isolated, experience greater APDs, a racial or ethnic minority, less educated, lower income, less physically active, smokers, and heavy drinkers. However, there were no significant differences in job strain, prevalence of MDE, or hypertension prevalence at baseline (details available upon request). Finally, while these results offer promising evidence on ACEs and APDs for hypertension incidence, they raise more questions regarding the role of these stress exposures in the broader context of cardiometabolic health. Hence, the contributions of ACEs and APDs to manifested diseases and relevant biomarkers deserve further investigation.

## 5. Conclusions

In a national, population-based cohort study of U.S. workers, APDs at baseline were prospectively associated with increased risk of hypertension within 20 years of follow-up. Elevated risk of hypertension incidence by APDs exposure was stronger when ACEs were present. As hypertension is a main driver of coronary events and cardiovascular deaths, future research on interplay between ACEs and APDs in relation to cardiovascular health outcomes are warranted.

## Figures and Tables

**Table 1:** Characteristics of the Sample Population at MIDUS I (*n* = 2568).

Variables (*n*, %)	
Mean age (SD)	42.89 (10.48)
Sex	
Male	1301 (50.66)
Female	1267 (49.34)
Race	
White	2388 (92.99)
Black	82 (3.19)
Other	98 (3.82)
Educational attainment	
University or more	1033 (40.23)
Some college	784 (30.53)
High school or less	751 (29.24)
Household income (annual USD)	
<45,000	883 (34.38)
45,000–89,999	928 (36.14)
≥90,000	757 (29.48)
Smoking status	
No	2034 (79.21)
Yes	534 (20.79)
Alcohol consumption	
Low to moderate drinking	2437 (94.90)
Heavy drinking	131 (5.10)
Physical activity	
High	1842 (71.73)
Moderate	491 (19.12)
Low	235 (9.15)
Major depressive episode	
No	2283 (88.90)
Yes	285 (11.10)
Adverse childhood experiences	
Low	1576 (61.37)
High	992 (38.63)
Adulthood psychosocial disadvantages	
Low	1733 (67.48)
Moderate	742 (28.89)
High	93 (3.62)
Incident hypertension	
No	1634 (63.63)
Yes	934 (36.37)

**Table 2:** Associations of ACEs, APDs at MIDUS I with Incident Hypertension across MIDUS II and III (HRs and 95% CIs) (*n* = 2568).

	Number of Exposed Participants (Number of Incident Hypertension Cases)	Incidence Rate of Hypertension (Per 1000 Person Years)	Model 0	Model I	Model II	Model III	Model IV
ACEs							
Low	1576 (562)	26.05	1.00	1.00	1.00	1.00	1.00
High	992 (372)	27.73	1.06 (0.93, 1.21)	1.03 (0.90, 1.17)	0.99 (0.86, 1.13)	0.96 (0.84, 1.11)	0.96 (0.84, 1.10)
APDs							
Low	1733 (607)	25.53	1.00	1.00	1.00	1.00	1.00
Moderate	742 (281)	28.14	1.11 (0.96, 1.28)	1.20 (1.04, 1.39) *	1.17 (1.02, 1.35) *	1.15 (0.99, 1.32)	1.14 (0.99, 1.32)
High	93 (46)	37.40	1.48 (1.10, 2.00) *	1.66 (1.23, 2.24) **	1.55 (1.15, 2.11) **	1.48 (1.09, 2.01) *	1.48 (1.09, 2.01) *

ACEs: adverse childhood experiences; APDs: adulthood psychosocial disadvantages; CI, confidence interval; HR, hazard ratio. Cox proportional hazards regression, * *p* < 0.05, ** *p* < 0.01. Model 0: non-adjustment. Model I: adjustment for age and sex at baseline. Model II: Model I + additional adjustment for race, educational attainment, and household income at baseline. Model III: Model II + additional adjustment for smoking, alcohol consumption, and physical exercise at baseline. Model IV: Model III + additional adjustment for major depressive episode at baseline.

**Table 3:** Associations of APDs at MIDUS I with Incident Hypertension across MIDUS II and III, Stratified by ACEs (HRs and 95% CIs) (*n* = 2568).

	Number of Exposed Participants (Number of Incident Hypertension Cases)	Incidence Rate of Hypertension (Per 1000 Person Years)	Model 0	Model I	Model II	Model III	Model IV
ACEs (low) (*n* = 1576)							
APDs							
Low	1103 (386)	25.52	1.00	1.00	1.00	1.00	1.00
Moderate	422 (153)	26.58	1.05 (0.87, 1.26)	1.14 (0.94, 1.37)	1.10 (0.91, 1.33)	1.08 (0.89, 1.30)	1.08 (0.89, 1.30)
High	51 (23)	33.09	1.29 (0.85, 1.96)	1.49 (0.98, 2.28)	1.43 (0.93, 2.19)	1.35 (0.88, 2.08)	1.34 (0.87, 2.06)
ACEs (high) (*n* = 992)							
APDs							
Low	630 (221)	25.95	1.00	1.00	1.00	1.00	1.00
Moderate	320 (128)	30.27	1.21 (0.97, 1.50)	1.30 (1.04, 1.61) *	1.29 (1.03, 1.61) *	1.27 (1.01, 1.59) *	1.27 (1.01, 1.60) *
High	42 (23)	43.00	1.76 (1.14, 2.70) *	1.88 (1.22, 2.90) **	1.81 (1.16, 2.81) **	1.83 (1.17, 2.85) **	1.83 (1.17, 2.86) **

ACEs: adverse childhood experiences; APDs: adulthood psychosocial disadvantages; CI, confidence interval; HR, hazard ratio. Cox proportional hazards regression, * *p* < 0.05, ** *p* < 0.01. Model 0: non-adjustment. Model I: adjustment for age and sex at baseline. Model II: Model I + additional adjustment for race, educational attainment, and household income at baseline. Model III: Model II + additional adjustment for smoking, alcohol consumption, and physical exercise at baseline. Model IV: Model III + additional adjustment for major depressive episode at baseline.

## Data Availability

Data used in this study are publicly available at https://www.icpsr.umich.edu/web/ICPSR/series/203 (Accessed on 28th May 2021).

## Appendix A

**Table A1:** Characteristics of the Sample Population at MIDUS I (*n* = 2037).

Variables (*n*, %)	
Mean age (SD)	42.12 (10.35)
Sex	
Male	980 (48.11)
Female	1057 (51.89)
Race	
White	1888 (92.69)
Black	70 (3.44)
Non-white	79 (3.88)
Educational attainment	
University or more	817 (40.11)
Some college	608 (29.85)
High school or less	612 (30.04)
Household income (annual USD)	
<45,000	683 (33.53)
45,000–89,999	736 (36.13)
≥90,000	618 (30.34)
Smoking status	
No	1610 (79.04)
Yes	427 (20.96)
Alcohol consumption	
Low to moderate drinking	1943 (95.39)
Heavy drinking	94 (4.61)
Physical activity	
High	1482 (72.75)
Moderate	371 (18.21)
Low	184 (9.03)
Major depressive episode	
No	1815 (89.10)
Yes	222 (10.90)
Adverse childhood experiences	
Low	1249 (61.32)
High	788 (38.68)
Adulthood psychosocial disadvantages	
Low	1356 (66.57)
Moderate	604 (29.65)
High	77 (3.78)
Incident hypertension	
No	1351 (66.32)
Yes	686 (33.68)

**Table A2:** Associations of ACEs, APDs at MIDUS I with Incident Hypertension across MIDUS II and III (HRs and 95% CIs) (*n* = 2037).

	Number of Exposed Participants (Number of Incident Hypertension Cases)	Incidence Rate of Hypertension (Per 1000 Person Years)	Model 0	Model I	Model II	Model III	Model IV
ACEs							
Low	1249 (400)	23.01	1.00	1.00	1.00	1.00	1.00
High	788 (286)	26.66	1.16 (0.99, 1.35)	1.13 (0.97, 1.31)	1.07 (0.92, 1.25)	1.04 (0.89, 1.22)	1.04 (0.89, 1.22)
APDs							
Low	1356 (431)	22.88	1.00	1.00	1.00	1.00	1.00
Moderate	604 (216)	26.21	1.14 (0.97, 1.35)	1.20 (1.02, 1.42) *	1.18 (1.00, 1.40) *	1.16 (0.98, 1.37)	1.15 (0.98, 1.36)
High	77 (39)	37.97	1.67 (1.20, 2.32) **	1.80 (1.30, 2.50) **	1.71 (1.22, 2.39) **	1.62 (1.15, 2.27) **	1.61 (1.15, 2.26) **

ACEs: adverse childhood experiences; APDs: adulthood psychosocial disadvantages; CI, confidence interval; HR, hazard ratio. Cox proportional hazards regression, * *p* < 0.05, ** *p* < 0.01. Model 0: non-adjustment. Model I: adjustment for age and sex at baseline. Model II: Model I + additional adjustment for race, educational attainment, and household income at baseline. Model III: Model II + additional adjustment for smoking, alcohol consumption, and physical exercise at baseline. Model IV: Model III + additional adjustment for major depressive episode at baseline.

**Table A3:** Associations of APDs at MIDUS I with Incident Hypertension across MIDUS II and III, Stratified by ACEs (HRs and 95% CIs) (*n* = 2037).

	Number of Exposed Participants (Number of Incident Hypertension Cases)	Incidence Rate of Hypertension (Per 1000 Person Years)	Model 0	Model I	Model II	Model III	Model IV
ACEs (low) (*n* = 1249)							
APDs							
Low	871 (274)	25.16	1.00	1.00	1.00	1.00	1.00
Moderate	336 (108)	27.98	1.03 (0.83, 1.29)	1.11 (0.89, 1.39)	1.09 (0.87, 1.36)	1.06 (0.84, 1.33)	1.06 (0.84, 1.32)
High	42 (18)	36.57	1.39 (0.86, 2.24)	1.54 (0.95, 2.48)	1.49 (0.92, 2.41)	1.38 (0.85, 2.24)	1.36 (0.83, 2.22)
ACEs (high) (*n* = 788)							
APDs							
Low	485 (157)	23.51	1.00	1.00	1.00	1.00	1.00
Moderate	268 (108)	30.03	1.30 (1.01, 1.66) *	1.34 (1.05, 1.72) *	1.36 (1.06, 1.76) *	1.35 (1.04, 1.74) *	1.35 (1.04, 1.75) *
High	35 (21)	46.37	2.07 (1.31, 3.63) **	2.14 (1.35, 3.37) **	2.04 (1.27, 3.27) **	2.11 (1.31, 3.39) **	2.11 (1.31, 3.40) **

ACEs: adverse childhood experiences; APDs: adulthood psychosocial disadvantages; CI, confidence interval; HR, hazard ratio. Cox proportional hazards regression, * *p* < 0.05, ** *p* < 0.01. Model 0: non-adjustment. Model I: adjustment for age and sex at baseline. Model II: Model I + additional adjustment for race, educational attainment, and household income at baseline. Model III: Model II + additional adjustment for smoking, alcohol consumption, and physical exercise at baseline. Model IV: Model III + additional adjustment for major depressive episode at baseline.
